# Psychological distress among infectious disease physicians during the response to the COVID-19 outbreak in the Republic of Korea

**DOI:** 10.1186/s12889-020-09886-w

**Published:** 2020-11-27

**Authors:** Se Yoon Park, Bongyoung Kim, Dong Sik Jung, Sook In Jung, Won Sup Oh, Shin-Woo Kim, Kyong Ran Peck, Hyun-Ha Chang, The Korean Society of Infectious Diseases

**Affiliations:** 1 Division of Infectious Diseases, Department of Internal Medicine, Soonchunhyang University Seoul Hospital, Soonchunhyang University College of Medicine, Seoul, Republic of Korea; 2 Department of Internal Medicine, Hanyang University College of Medicine, Seoul, Republic of Korea; 3 Division of Infectious Diseases, Department of Internal Medicine, Dong-A University College of Medicine, Busan, Republic of Korea; 4 Department of Infectious Diseases, Chonnam National University Medical School, Gwangju, Republic of Korea; 5 Division of Infectious Diseases, Department of Internal Medicine, Kangwon National University Hospital, Kangwon National University School of Medicine, Chuncheon, Republic of Korea; 6 Division of Infectious Diseases, Department of Internal Medicine, School of Medicine, Kyungpook National University, 130 Dongdeok-ro, Daegu, Jung-gu 41944 South Korea; 7 Division of Infectious Diseases, Samsung Medical Center, Sungkyunkwan University School of Medicine, Seoul, Republic of Korea

**Keywords:** COVID-19, Infectious diseases medicine, Burnout, Psychological, Psychological distress, South Korea

## Abstract

**Background:**

This study aimed to investigate psychological distress among infectious disease (ID) physicians during the coronavirus disease 2019 (COVID-19) outbreak in the Republic of Korea.

**Methods:**

Using an online-based survey link sent via text message and email, we conducted a survey from April 21 to 25, 2020, targeting all ID physicians currently working in ID (*n* = 265). The questionnaire was based on the Maslach Burnout Inventory-Human Services Survey and the Depression, Anxiety, and Stress Scales, and information was collected on factors protecting against psychological distress and difficulties in relation to COVID-19.

**Results:**

Of 265 ID physicians, 115 (43.3%) responded, showing burnout (97, 90.4%), depression (20, 17.4%), anxiety (23, 20.0%), and stress (5, 4.3%). There were no differences in terms of distress between ID physicians who were directly involved in the care of patients with COVID-19 or not. Greater than 50% of physicians valued their work and felt recognized by others, whereas < 10% indicated that sufficient human and financial support and private time had been provided during the outbreak. The most challenging issues concerned a lack of attending physicians caring for COVID-19 patients or infection control practitioners, a shortage of personal protective equipment or airborne infection isolation rooms, pressure for research, and lack of guidelines for COVID-19 management.

**Conclusions:**

During the COVID-19 outbreak in the Republic of Korea, most respondents reported psychological distress. Preparing strategies to secure human resources are crucial to prepare effectively for future epidemics and pandemics.

**Supplementary Information:**

The online version contains supplementary material available at 10.1186/s12889-020-09886-w.

## Background

An infectious diseases (ID) physician has an important role when dealing with emerging diseases such as coronavirus disease 2019 (COVID-19). ID physicians have a combined role in participating in patient care and providing advice based on scientific evidence for prevention strategies to each medical institution and government [1]. Since the 2000s, the Republic of Korea (ROK) has experienced several ID outbreaks, including the H1N1 influenza pandemic in 2009 and the Middle East respiratory syndrome (MERS) outbreak in 2015 [1, 2].

The first case of COVID-19 in the ROK was diagnosed in a person who entered from Wuhan, China, in January 2020 and, since then, the ROK has been combatting the COVID-19 pandemic. A large-scale outbreak occurred in Daegu and surrounding areas that was linked to a religious event held in mid-February, followed by several smaller outbreaks throughout the ROK and, by the end of April 2020, a total of 10,752 patients with COVID-19 had been confirmed, along with 244 COVID-19-related deaths [3].

In response to the COVID-19 outbreak that has continued for more than three months, the psychological effects of this outbreak on medical staff has been analyzed in several countries [4, 5]. However, there has been no analysis of the psychological effects on ID physicians, specifically, specialists who have played a pivotal role in responding to the COVID-19 outbreak in many countries, including the ROK. This study aimed to analyze the extent of psychological distress among ID physicians in the ROK during the COVID-19 outbreak. Moreover, we aimed to investigate factors protecting against psychological distress and the difficulties facing ID physicians when dealing with the COVID-19 outbreak to determine their specific work-related requirements.

## Methods

### Study design and population

This study used a cross-sectional quantitative design. A survey was conducted over a 5-day period, from April 21 to 25, 2020, targeting all ID physicians in the ROK (*n* = 275). At the time of the survey, 10 ID physicians were identified as either having retired or died and were excluded. In total, 265 ID physicians were identified as potential participants in the survey and an online-based survey link was forwarded to them via text messaging and e-mail. No financial incentive was offered for completion of the questionnaire. The respondents were anonymized and were requested to enter their own identification number to distinguish any duplicated answers. The study protocol was approved by the Institutional Review Board (IRB) of Soonchunhyang University Seoul Hospital. Written informed consent was obtained from participants via their online participation.

### Survey items

The questionnaire was based on the Maslach Burnout Inventory-Human Services Survey (MBI-HSS) and the Depression, Anxiety, and Stress Scales (DASS-21). The MBI-HSS is used to assess emotional exhaustion, depersonalization, and lack of personal accomplishment. The license provided by Mind Garden® was obtained for the use of the Maslach Burnout Inventory. Summed scores are compared with standardized thresholds set out in the MBI-HSS manual (cutoff scores: > 22 for emotional exhaustion, > 7 for depersonalization, < 36 for personal accomplishment) [6]. Overall burnout is defined as a high score in either the emotional exhaustion or depersonalization subscale [7]. The DASS-21 is a 21-item system that provides independent measures of depression, stress, and anxiety with recommended severity thresholds. Cutoff scores > 9, > 7, and > 14 indicate a positive screen for depression, anxiety, and stress, respectively [8].

We investigated factors protecting against psychological distress among ID physicians. We used a survey item with a 5-point Likert scale by modifying questions of the ID Burnout Inventory from a previous study [7] and divided the questionnaire into four categories, as follows: (1) value of work and recognition from others, (2) human and financial support, (3) housework and childcare, and (4) ensuring sufficient private time. We also investigated difficulties experienced by ID physicians when responding to the COVID-19 outbreak. A respondent could select up to three items among 11 items concerning difficulties they had been experiencing (see the questionnaire in Supplementary).

### Statistical analysis

SPSS version 24.0 for Windows (IBM; Armonk, NY) was used for statistical analysis. Chi-squared or Fisher’s exact tests were used to compare categorical variables, and Student’s t-test was used for continuous variables. Variables with a *P*-value *<* 0.05 were considered statistically significant.

## Results

### Baseline characteristics of the ID physicians

Of 265 surveys, we received 115 (43.3%) responses. The respondents’ characteristics are shown in Table 1. All respondents were working in hospitals; 53 (46.1%) and 48 (41.7%) respondents were working as Directors of ID Departments or as Directors of an Infection Control team, respectively. The median number of years spent working as a Director of an Infection Control team was 5 years (interquartile range [IQR], 3–13 years). COVID-19-related work was reported to comprise > 80% of total workload for 23 (20.0%) and 67 (58.3%) respondents for the last two weeks and the busiest week of the COVID-19 outbreak, respectively. The mean number of weekly working hours was 62 h in April 2020 and 84 h during the busiest week of the COVID-19 outbreak. The ID physicians who were treating patients with COVID-19 worked significantly longer (*P* < 0.001).

**Table 1:** Baseline characteristics of the ID physicians

Characteristics	Total, *n* = 115 (%)	COVID-19 patient care		*P-*value
		Yes (*n* = 78)	No (*n* = 37)	
Sex, Female	67 (58.3)	43 (55.1)	24 (64.9)	0.323
Age, median (IQR), years	41 (37–48)	42 (37–48)	41 (38–47)	0.904
Marital status				
Married	94 (81.7)	66 (84.6)	28 (75.7)	0.246
Have children	86 (74.8)	62 (79.5)	24 (64.9)	0.092
Position in hospital				
Director of an ID department	53 (46.1)	33 (42.3)	20 (54.1)	0.238
Director of an Infection Control team	48 (41.7)	28 (35.9)	20 (54.1)	0.065
Both	30 (26.1)	13 (16.7)	17 (45.9)	0.001
Numbers of ID specialists in the hospital, median (IQR)	3 (2–4)	3 (2–4)	2 (1–3)	0.194
Length of career as an ID specialist (IQR), years	8 (4–13)	9 (3–13)	7 (4–14)	0.817
Type of hospital				< 0.001
University-affiliated hospital	86 (74.8)	65 (83.3)	21 (56.8)	
Private, non-university-affiliated hospital	19 (16.5)	5 (6.4)	15 (40.5)	
Public, non-university-affiliated hospital	9 (7.8)	8 (10.3)	1 (2.7)	
Number of hospital beds				< 0.001
≥ 1200	22 (19.1)	16 (20.5)	6 (16.2)	
900–1200	21 (18.3)	21 (26.9)	0	
600–900	45 (39.1)	29 (37.2)	16 (43.2)	
300–600	24 (20.9)	12 (15.4)	12 (32.4)	
< 300	3 (2.6)	0	3 (8.1)	
Participation in COVID-19 related night duty	68 (59.1)	56 (71.8)	12 (32.4)	< 0.001
Working hours per week				
Recent 2 weeks (April 2020), median (IQR), h	62 (50–74)	65 (54–76)	52 (45–62)	< 0.001
During COVID-19 outbreak, median (IQR), h	84 (66–102)	90 (75–115)	70 (53–84)	< 0.001

*Abbreviations*: *COVID-19* coronavirus disease-19, *ID* infectious disease, *IQR* interquartile range

### Results concerning the psychological effects of COVID-19

Ninety-seven (90.4%) respondents screened positive for burnout, 20 (17.4%) for depression, 23 (20.0%) for anxiety, and five (4.3%) for stress (Table 2). There was no difference among ID physicians who cared for patients with or without COVID-19 (Table 2). Prevalence rates for burnout were similar between the sexes. Female ID physicians tended to report depression, anxiety, and stress more frequently than their male counterparts, and a higher proportion of female ID physicians were found to endorse emotional exhaustion (Supplemental Table 1).

**Table 2:** Prevalence of depression, anxiety, stress, burnout and mean DASS-21/MBI-HSS scores according to care of patients with COVID-19

Outcome	Total (*n* = 115)		COVID-19 patient care, Yes (*n* = 78)		COVID-19 patient care, No (*n* = 37)		*P-*value
	Prevalence, *n* (%)	Score, mean ± SD	Prevalence, *n* (%)	Score, mean ± SD	Prevalence, *n* (%)	Score, mean ± SD	
DASS-21							
Depression	20 (17.4)	5.45 ± 4.16	14 (17.9)	5.58 ± 4.30	6 (16.2)	5.19 ± 3.90	0.643
Anxiety	23 (20.0)	3.88 ± 3.74	17 (21.8)	3.85 ± 3.63	6 (16.2)	3.95 ± 4.01	0.894
Stress	5 (4.3)	6.23 ± 3.86	4 (5.1)	6.21 ± 3.94	1 (2.7)	6.30 ± 3.75	0.905
MBI-HSS scale							
Emotional exhaustion	97 (84.3)	34.92 ± 10.01	68 (87.2)	35.96 ± 10.32	29 (78.4)	32.73 ± 9.25	0.108
Depersonalization	76 (66.1)	10.55 ± 5.69	51 (65.4)	10.71 ± 6.01	25 (67.6)	10.22 ± 5.01	0.669
Personal accomplishments	76 (66.1)	31.66 ± 8.18	51 (65.4)	32.21 ± 8.54	25 (67.6)	30.51 ± 7.34	0.302
Burnout	104 (90.4)	NA	71 (91.0)	NA	33 (89.2)	NA	0.774

*Abbreviations*: *DASS-21* Depression, Anxiety, and Stress scale-21, *MBI-HSS* the Maslach Burnout Inventory-Human Services Survey, *NA* not available, *SD* standard deviation

**Fig. 1:**
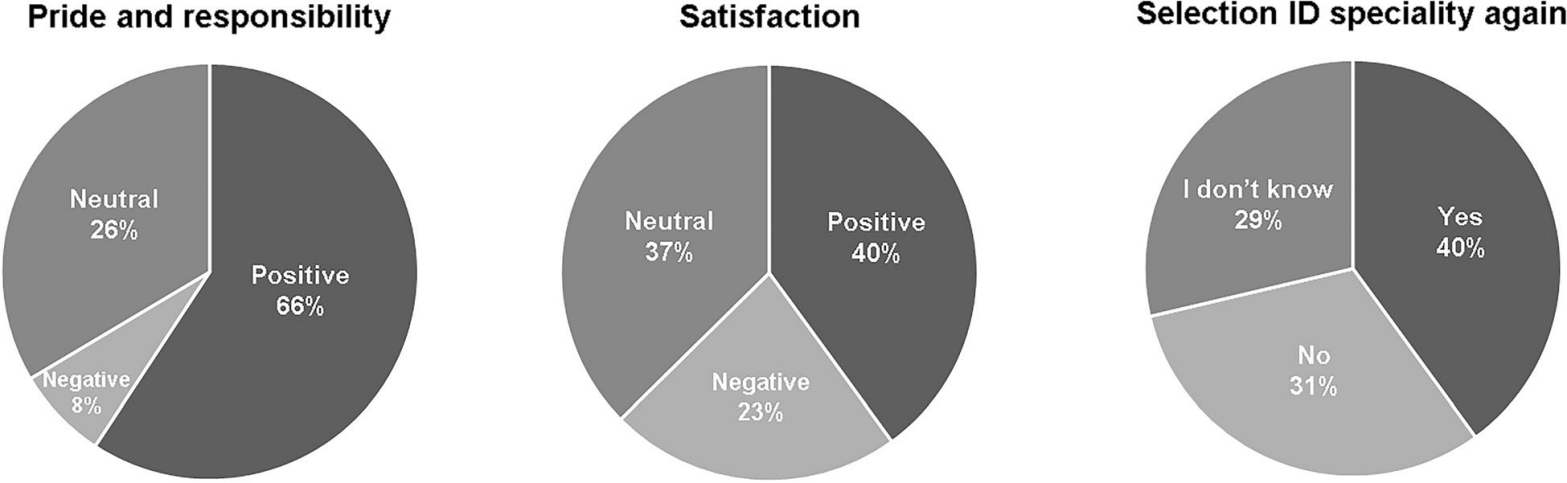
Pride and responsibility, and satisfaction as an infectious disease physician, and intention to select infectious disease specialty again if responders were to have another opportunity to choose a specialty

Forty-six (40%) respondents reported that they were satisfied working as ID physicians, whereas 26 (22.6%) reported they were not satisfied. When asked if they would choose an ID specialty if they could choose their specialty again, 46 (40%) responded positively and 36 (31.3%) responded negatively. Sixty-seven (76%) respondents reported a sense of responsibility and pride in performing COVID-19-related tasks, whereas nine (6.1%) did not (Fig. 1). In female ID physicians, satisfaction was significantly lower than that in male ID physicians (32.8% vs. 50%, *p* = 0.018). There was no difference in re-selection of an ID specialty (31.1% vs. 52.1%, *p* = 0.072) or pride and responsibility (67.2% vs. 64.2%, *p* = 0.018).

### Factors protecting against psychological distress

More than 50% of respondents reported that their work was valued and that it had been recognized by others. Less than 20% of ID physicians reported that they had sufficient human or financial support (in terms of adequate staff [16.5%], financial compensation [9.6%], work-life balance [8.7%], and adequate coverage of responsibility [7.8%]), and sufficient private time (i.e., working at home was not required [2.6%] and they had enough time to enjoy leisure activities and time spent at home [2.6%]). Only 33% of respondents reported sharing household responsibilities equally with their spouse/partner and 15.7% of respondents reported that childcare was not a significant source of stress for them (Table 3).

**Table 3:** Factors protective against burnout as an ID physician

Categories	*Total n=115, n* (%)
Value of work and recognition from others	
I feel my professional opinions are valued by other physicians	102 (88.7)
I feel that my spouse or partner values my work	81 (70.4)
I feel that my career is a large part of my identity as an adult	80 (69.6)
I feel that my contributions are adequately recognized and acknowledged by my supervisors	67 (58.3)
Human and financial support	
I feel that I have adequate support staff for maximum productivity in this role	19 (16.5)
I feel that I am adequately financially compensated for my work	11 (9.6)
I feel that it is possible to balance work and non-work responsibilities	10 (8.7)
I feel that I have adequate coverage of my work responsibilities to tend to personal matters, emergencies, illness, etc.	9 (7.8)
Housework and childcare	
My spouse/partner and I try our best to share household responsibilities equally	38 (33)
Childcare is not a significant source of stress for me	18 (15.7)
Ensuring enough private time	
I do not often have to complete work at home (clinician, infection control, and research)	3 (2.6)
I have enough time to do something I enjoy	3 (2.6)

### Difficulties in responding to the COVID-19 outbreak

**Fig. 2:**
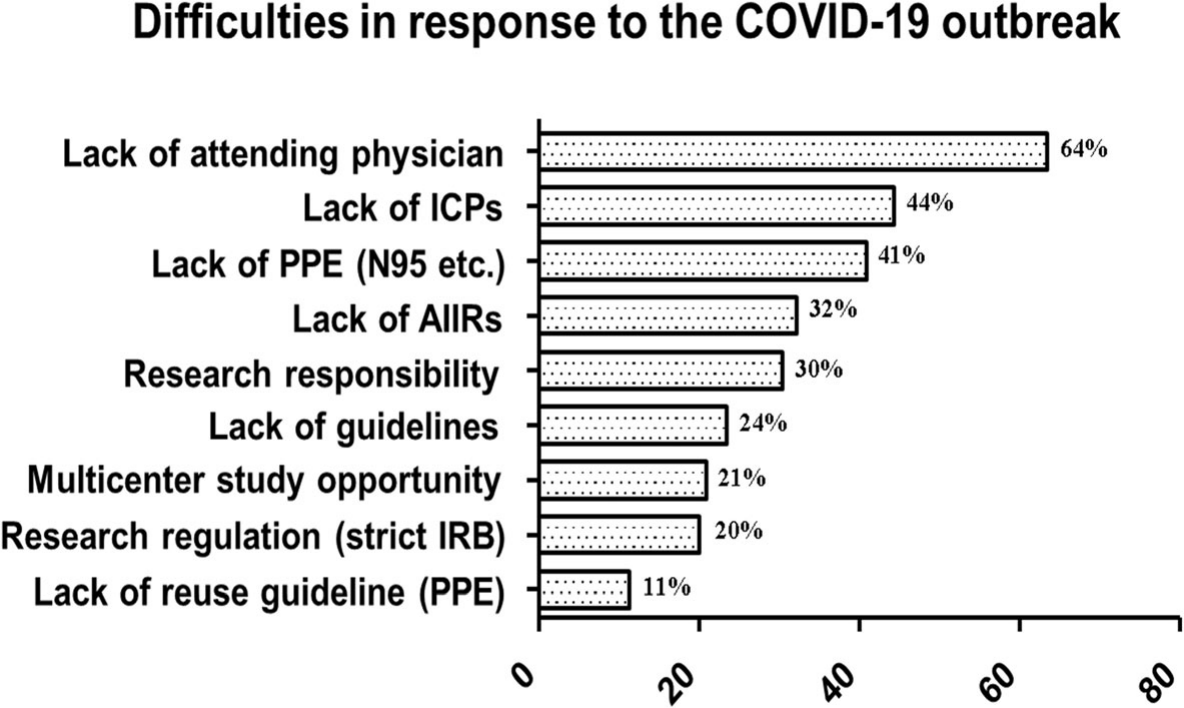
Difficulties in response to the COVID-19 outbreak. Abbreviations: COVID-19, coronavirus disease 19; ICPs, infection control practitioners; PPE, personal protective equipment, AIIRs, airborne infection isolation rooms; IRB, Institutional Review Board

ID physicians most commonly reported difficulties derived from a lack of healthcare workers and infection control practitioners, followed by a lack of personal protective equipment (PPE), airborne-infection isolation rooms (AIIRs), research support (responsibility, multicenter opportunities, and strict IRB requirements), as well as guidelines for COVID-19-related care and PPE reuse (Fig. 2).

## Discussion

During the COVID-19 outbreak, the prevalence rate of burnout among the ID physicians in the ROK responding to our survey was 90.4%. There have been inconsistencies in defining burnout and in the assessment methods used to identify burnout across studies; however, our results showed higher burnout than those of a previous meta-analysis involving physicians, which reported a burnout range from 0 to 80.5% [9]. Compared to studies conducted in 1992 and in 2008 in the United States involving ID physicians that reported burnout levels of 40–50%, the prevalence of burnout in our study was considerably higher [7, 10]. Our results showed that ID physicians experienced burnout more during the COVID-19 outbreak. One study, undertaken during the COVID-19 pandemic at an oncology ward in Wuhan, China, reported the prevalence of burnout among oncology physicians and nurses was 26%. Unexpectedly, the prevalence was significantly lower for frontline medical personnel than for those working in the oncology ward [5]. In our study, direct COVID-19 patient care resulted in long working hours and night duty; however, the frequency of burnout and the levels of depression, anxiety, and stress did not differ significantly between ID physicians who cared for patients with COVID-19 and those who did not. Moreover, female ID physicians were found to report a significantly higher frequency of psychological issues in depression, anxiety, stress, and emotional exhaustion compared to males. It is possible that they may be at risk because of undertaking greater responsibilities for childcare and household duties due to social changes occurring at the start of the pandemic, such as the closure of schools and delays in their re-opening.

In November 2019, there were 255 ID physicians working in the ROK, who comprised only 3.2% of subspecialized internal medicine doctors. The number of ID physicians was 0.5 per 100,000 people, which is a lower ratio than the number of ID physicians working in the United States (2.8 per 100,000 people) [11]. ID physicians play an important role not only in the outbreak response to COVID-19 but also in direct patient care, infection control, antibiotic stewardship, education, disease surveillance, and outpatient antibiotic therapy [12, 13]. Concerning patient care, intervention by ID physicians has been reported to be significantly associated with lower mortality rates, shorter hospital stays, and reduced healthcare costs [14, 15]. Many studies have shown that consultation with ID physicians can optimize antibiotic prescription [16, 17]. In a study conducted in a large Korean hospital, one ID specialist-led antimicrobial stewardship program concerning antibiotic use resulted in a meaningful reduction in antibiotic use and a decrease in the antibiotic resistance rate without changing the mortality rate [17]. However, despite the proven efficacy of ID physician intervention, much of their work has been reported to be undercompensated [18].

During the COVID-19 outbreak, most respondents felt valued in their work and had been recognized by others. However, > 90% of respondents considered that there was a shortage of adequate staff and inadequate financial compensation. Supporting medical staff and infection control practitioners who directly care for patients with COVID-19 was found to be a more urgent requirement than dealing with issues arising due to a lack of PPE or AIIRs. All respondents were working in hospitals, and more than two-thirds worked in university-affiliated hospitals. Furthermore, > 40% of the ID physicians were ID Department Directors or Directors of an Infection Control team. Our results showed that most ID physicians in the ROK played various roles, not only in terms of patient care but also in infection control, administration, education, and research.

The ROK experienced a MERS outbreak in 2015; however, only approximately 50 ID physicians had been subsequently trained by 2020, with only 10 applicants for the ID specialty annually (data not shown). During an infectious disease crisis, ID physicians have acted as coordinators and have been at the forefront of the response to the outbreak and in providing patient care, but they have been reported to be inadequately compensated by relevant institutions or governments [1]. This latter factor may have led to fewer applicants for the role of ID physician or as ICP. A shortage of ID physicians is likely to require greater participation in various duties related to caring for confirmed patients with COVID-19 and in infection control with a more demanding workload, resulting in a higher likelihood of psychological distress such as burnout, depression, anxiety, and stress.

In the survey, only 40% of the ID physicians responded that they were satisfied with their work and that they would select to be an ID physician again if they had another chance to choose their specialty. However, 76% of the respondents indicated they felt a sense of responsibility and pride in their work in combatting COVID-19. In particular, female ID physicians were found to have significantly lower satisfaction than males. This may also be related to the psychological distress reported as experienced by female ID physicians, which was worse than that reported for their male colleagues. One Korean study of gastrointestinal physicians highlighted a work-life imbalance, and burnout was found to be most severe among young female gastrointestinal physicians due to additional pressure to address domestic requirements [19]. In our study, this factor could also be a possible explanation for differences between male and female ID physicians, as female ID physicians reported feeling considerably more pressure than male ID physicians in terms of childcare commitments. Further research is needed to confirm whether this factor is important in relation to ID physicians. In addition, this apparent sex difference needs to be re-evaluated outside of the COVID-19 outbreak.

This study had some limitations. As only ID physicians were surveyed, it would be difficult to draw conclusions in terms of other specialties. Further, the response rate was only 43.3%, and the responses obtained may not be accurately representative of the ID physician population. Because of the relatively short response time, it is possible that busy respondents could not answer. However, it would have been challenging to attain a higher response rate, despite the survey time having been extended for > 5 days, because of the reality of time constraints and high workload volumes due to the COVID-19 outbreak during this survey period.

## Conclusions

The findings of our study showed that the COVID-19 outbreak imposed considerable psychological distress on ID physicians and that greater numbers of ID physicians and support staff were needed for patient care and infection control. Strategies to secure human resources are crucial to prepare effectively for future epidemics and pandemics.

## Supplementary Information

**Additional file 1:** **Supplemental Table 1.** Prevalence of depression, anxiety, stress, burnout and mean DASS-21/MBI-HSS scale according to sex.

## Data Availability

The analysis data is not available as it contains protected personal information. An anonymized data set is not available upon request.

## Abbreviations

ID: Infectious diseases
COVID-19: Coronavirus disease 2019
ROK: Republic of Korea
MERS: Middle East respiratory syndrome
IRB: Institutional Review Board
MBI-HSS: Maslach Burnout Inventory-Human Services Survey
DASS-21: Depression, Anxiety, and Stress Scales
IQR: Interquartile range
PPE: Personal protective equipment
AIIRs: Airborne-infection isolation rooms
