# Supplementary material for: Psychological distress among infectious disease physicians during the response to the COVID-19 outbreak in the Republic of Korea
Source: BMC Public Health. 2020 Nov 27;20:1811. doi: 10.1186/s12889-020-09886-w (PMC7691971; doi:10.1186/s12889-020-09886-w)
Supplement: Supplementary file 1 — Additional file 1: Supplemental Table 1. Prevalence of depression, anxiety, stress, burnout and mean DASS-21/MBI-HSS scale according to sex. [file 12889_2020_9886_MOESM1_ESM.docx]

**Additional file**

**Psychological distress among infectious disease physicians during the response to the COVID-19 outbreak in the Republic of Korea**

Se Yoon Park, Bongyoung Kim, Dong Sik Jung, Sook In Jung, Won Sup Oh, Shin-Woo Kim, Kyong Ran Peck, Hyun-Ha Chang: The Korean Society of Infectious Diseases

Contents:

Supplemental Table 1. Prevalence of depression, anxiety, stress, burnout and mean DASS-21/MBI-HSS scale according to sex ---------------------------------------------------------- p2

Questionnaire: a survey concerning the psychological distress due to the COVID-19 pandemic on infectious diseases physicians in the Republic of Korea ------------------- p4

**Supplemental Table 1** Prevalence of depression, anxiety, stress, burnout and mean DASS-21/MBI-HSS scale according to sex

| Outcome | Total (n = 115) | | Male (n = 48) | | Female (n = 67) | | *P-*value |
| --- | --- | --- | --- | --- | --- | --- | --- |
|  | Prevalence, n (%) | Score,  mean ± SD | Prevalence, n (%) | Score,  mean ± SD | Prevalence, n (%) | Score,  mean ± SD |  |
| DASS-21 |  |  |  |  |  |  |  |
| Depression | 20 (17.4) | 5.45 ± 4.16 | 3 (6.3) | 4.15 ± 3.40 | 17 (25.4) | 6.39 ± 4.42 | 0.003 |
| Anxiety | 23 (20.0) | 3.88 ± 3.74 | 6 (12.5) | 2.83 ± 3.42 | 17 (25.4) | 4.63 ± 3.80 | 0.011 |
| Stress | 5 (4.3) | 6.23 ± 3.86 | 1 (2.1) | 5.27 ± 3.61 | 4 (6.0) | 6.93 ± 3.91 | 0.023 |
| MBI-HSS scale |  |  |  |  |  |  |  |
| Emotional exhaustion | 97 (84.3) | 34.92 ± 0.01 | 38 (79.2) | 32.56 ± 10.36 | 59 (88.1) | 36.61 ± 9.57 | 0.033 |
| Depersonalization | 76 (66.1) | 10.55 ± 5.69 | 29 (60.4) | 9.69 ± 5.86 | 47 (70.1) | 11.16 ± 5.52 | 0.171 |
| Personal accomplishments | 76 (66.1) | 31.66 ± 8.18 | 33 (68.8) | 31.50 ± 8.44 | 43 (64.2) | 31.78 ± 8.05 | 0.859 |
| Burnout | 104 (90.4) | NA | 42 (87.5) | NA | 62 (92.5) | NA | 0.365 |

Abbreviations: DASS-21, Depression, Anxiety, and Stress scale-21; MBI-HSS, the Maslach Burnout Inventory-Human Services Survey; NA, not available; SD, standard deviation

**A survey concerning the psychological distress due to the COVID-19 pandemic on infectious diseases physicians in the Republic of Korea**

This survey will investigate the psychological distress experienced by infectious disease physicians and use the data for future measures and prevention. The survey is anonymous and without financial incentive. It is expected to take about 15 minutes, and participation of personnel other than the infectious diseases physicians (excluding fellows in training) is limited.

**0. Agreement to participate in the survey**
Do you agree to participate in this survey (a survey to investigate the psychological distress due to the COVID-19 pandemic on infectious diseases physicians in the Republic of Korea)?

□ Yes □ No

**1. Baseline information concerning the respondent and the affiliated hospital**

1-1. Sex □ Male □ Female

1-2. Age (________years)

1-3. Year of acquisition to the infectious diseases board? (___________)

1-4. Please indicate your position (multiple responses)

□ Director of the clinical department □ Director of Infection Control

□ Other assigned position in the hospital (___________) □ Not applicable

1-5. What type of medical institution do you work for?

□ Tertiary-care hospital □ Secondary-care hospital

□ Hospital □ Clinic

1-6. Which of the following is the medical institution where you work?

□ National University hospital □ Private University hospital

□ Public Hospital; Non-University hospital □ Private hospital; Non-University hospital

□ Other (___________)

1-7. Please select the size of the medical institution you work for.

□ <300 beds □ 300-600 beds □ 600-900 beds
□ 900-1200 beds □ ≥1200 beds

1-8. Does the hospital where you work run a state-designated negative-pressure isolation unit?

□ Yes □ No

1-9. Where is your work area?

□ Seoul □ Incheon □ Gyeonggi-do □ Busan

□ Daegu □ Ulsan □ Gyeongsangnam-do

□ Gyeongsangbuk-do □ Daejeon/Sejong □ Chungcheongnam-do

□ Chungcheongbuk-do □ Gwangju □ Jeollanam-do

□ Jeollabuk-do □ Gangwon-do □ Jeju-do

1-10. How many infectious diseases specialists are there in the hospital where you work? (___________)

1-11. How many confirmed patients with COVID-19 have you treated so far as an attending physician or physician? (including patients who have been dispatched to a living and treatment support center or another institution)

□ <10 □ 10-29 □ 30-49 □ 50-99
□ 100-199 □ 200-299 □ ≥300

**2. Duty status and hours of work**

2-1. Are you taking part in night-time or weekend care related to COVID-19?

□ Yes □ No

2-2. How many hours do you usually work a day?

| Working day | Total working hours | COVID-19 related working hours |
| --- | --- | --- |
| Weekday | ( ) Hours ( ) Minutes | ( ) Hours ( ) Minutes |
| Saturday | ( ) Hours ( ) Minutes | ( ) Hours ( ) Minutes |
| Sunday | ( ) Hours ( ) Minutes | ( ) Hours ( ) Minutes |
| Holidays (excluding Saturdays and Sundays) | ( ) Hours ( ) Minutes | ( ) Hours ( ) Minutes |

2-3. What is the percentage of daily work related to COVID-19?

□ ≤20% □ 21-40% □ 41-60% □ 61-80% □ 81-100%

**3. Motivation and satisfaction**

3-1. Do you have a feeling of pride or responsibility in your current COVID-19 related work? (patient care, infection control, etc.)

□ Yes □ No □ I don’t know

3-2. Are you satisfied with the work you are doing?

□ Yes □ No □ I don’t know

3-3. Considering the fact that you are dealing with infectious disease epidemic situations such as COVID-19, if you were able to choose your specialty again, would you choose infectious diseases again?

□ Yes □ No □ I don’t know

3-4. The following are some protective factors against psychological distress as an infectious physician. Please answer each question.

1 - I disagree. 2 - I partially disagree, 3 – Neutral, 4 – I agree to a certain extent, 5 – I totally agree

| I feel my professional opinions are valued by other physicians | 1 | 2 | 3 | 4 | 5 |
| --- | --- | --- | --- | --- | --- |
| I feel that my contributions are adequately recognized and acknowledged by my supervisors | 1 | 2 | 3 | 4 | 5 |
| I feel that I am adequately financially compensated for my work | 1 | 2 | 3 | 4 | 5 |
| I feel that I have adequate support staff for maximum productivity in this role | 1 | 2 | 3 | 4 | 5 |
| I feel that it is possible to balance work and non-work responsibilities | 1 | 2 | 3 | 4 | 5 |
| I do not often have to complete work at home (clinician, infection control, and research) | 1 | 2 | 3 | 4 | 5 |
| I feel that I have adequate coverage of my work responsibilities to tend to personal matters, emergencies, illness, etc. | 1 | 2 | 3 | 4 | 5 |
| I feel that my spouse or partner values my work | 1 | 2 | 3 | 4 | 5 |
| Childcare is not a significant source of stress for me | 1 | 2 | 3 | 4 | 5 |
| My spouse/partner and I try our best to share household responsibilities equally | 1 | 2 | 3 | 4 | 5 |
| I have enough time to do something I enjoy | 1 | 2 | 3 | 4 | 5 |
| I feel that my career is a large part of my identity as an adult | 1 | 2 | 3 | 4 | 5 |

**4. Difficulty in responding COVID-19, COVID-19 related burnout, depression, anxiety, and stress**

4-1. Do you fear the possibility of spreading COVID-19 to your family or colleague because of you?

□ Not at all □ It's not quite like that □ Neutral, □ I do a little bit □ Most of the time

4-2. Please select three items for the areas where you find the most difficult to deal with COVID-19 (patient care, infection control, research, etc).

□ Lack of attending physician who cares for COVID-19 patients

□ Lack of airborne infection isolation room

□ Lack of COVID-19 related guideline

□ Lack of infection control team practitioners

□ Lack of personal protective equipment

□ Lack of guideline about reuse personal protective equipment

□ Difficulty in linking basic research

□ Missing sample storage location

□ Research regulation (IRB, etc.)

□ Difficulty in multicenter study

□ Pressure of research

4-3. Please answer the following questions (Maslach Burn Inventory, MBI measurement tools). Please take into account the situation within a month based on the survey response date.

Please see the following reference:

*Maslach C, Jackson SE, Michael P. Leiter. Maslach Burnout Inventory Manual. 4th ed. https://www.mindgarden.com//117-maslach-burnout-inventory.*

4-4. Please answer the following questions (depression, anxiety and stress, DASS-21). Please take into account the situation within a month, based on the survey response date.

Please see the following reference:

*Lovibond SH, Lovibond PF. Manual for the depression anxiety stress scales. Sydney, N.S.W. : Psychology Foundation of Australia, c1995.*
